# Cinnamaldehyde Resist *Salmonella* Typhimurium Adhesion by Inhibiting Type I Fimbriae

**DOI:** 10.3390/molecules27227753

**Published:** 2022-11-10

**Authors:** Lizi Yin, Yuyun Dai, Han Chen, Xuewen He, Ping Ouyang, Xiaoli Huang, Xiangang Sun, Yanru Ai, Siyuan Lai, Ling Zhu, Zhiwen Xu

**Affiliations:** 1 College of Veterinary Medicine, Sichuan Agriculture University, Huimin Road 211, Chengdu 611130, China; 2 College of Animal Science and Technology, Sichuan Agriculture University, Huimin Road 211, Chengdu 611130, China

**Keywords:** cinnamaldehyde, *S*. typhimurium, type I fimbriae, proteomics

## Abstract

*Salmonella* Typhimurium (*S*. Typhimurium), a common foodborne pathogen, severely harms the public and food security. Type I fimbriae (T1F) of *S*. Typhimurium, plays a crucial role in the pathogenic processes; it mediates the adhesion of bacteria to the mannose receptor on the host cell, assists the bacteria to invade the host cell, and triggers an inflammatory response. Cinnamaldehyde is the main ingredient in cinnamon essential oil. In this study, cinnamaldehyde was demonstrated to inhibit the expression of T1F by hemagglutination inhibition test, transmission electron microscopy, and biofilms. The mechanism of cinnamaldehyde action was studied by proteomics technology, PCR and Western blotting. The results showed that cinnamaldehyde can inhibit T1F in *S*. typhimurium without the growth of bacteria, by regulating the level of expression and transcription of *fimA*, *fimZ*, *fimY*, *fimH* and *fimW*. Proteomics results showed that cinnamaldehyde downregulated the subunits and regulators of T1F. In addition, the invasion assays proved that cinnamaldehyde can indeed reduce the ability of *S*. typhimurium to adhere to cells. The results of animal experiments showed that the colonization in the intestinal tract and the expression levels of inflammatory cytokine were significantly decreased, and the intestinal mucosal immune factors MUC1 and MUC2 were increased under cinnamaldehyde treatment. Therefore, cinnamaldehyde may be a potential drug to target T1F to treat *Salmonella* infections.

## 1. Introduction

*Salmonella*, a gram-negative rod-shaped motile bacterium belonging to the Enterobacteriaceae family, can infect humans, domestic animals, poultry and other vertebrates Consequently, *Salmonella* infections have caused great economic losses and a great treatment burden globally [1,2]. Meanwhile, with the overuse and inappropriate usage of antibiotics, bacteria have developed resistance to almost all kinds of drugs. Therefore, new therapeutic strategies are urgently required for clinical treatment.

During its infection, *Salmonella* must resist or evade the host’s immune defense. Many pathogenic genes contribute to the survival of these bacteria in the host, including bacterial fimbriae, enterotoxin, virulence islands, etc. *Salmonella* establishes various strategies to adhere to host tissues by expressing an enormous number of fimbria, which play an important role in the adhesion of bacteria to epithelial cells and in the colonization of the small intestinal mucosa [3]. Fimbria is a fine structure on the surface of bacteria, composed primarily of the structural protein FimA. Molecular subunits are assembled by cytoplasmic chaperones and extracellular guides, which are very diverse, including *stg, sef*, *sta*, *ste*, *fim*, etc. [4]. Studies have shown that the loss of fimbriae leads to a decrease in the adhesion and invasion of *Salmonella* to epithelial cells [5]. *Salmonella typhimurium* has two distinct phenotypes: adherent and nonadherent. Adhesive cells were shown to produce type I fimbriae (T1F) that presumably were responsible for their adhesive properties, while non-adhesive cells did not [6]. T1F consists of fimA, fimH, fimF, fimZ, fimY, fimW in *Salmonella* [7]. T1F in *Salmonella enterica* serovar Typhimurium are proteinaceous surface appendages that carry adhesions specific for mannosylated glycoproteins. It mediates the adhesion of bacteria to the mannose receptor on the host cell, assists the bacteria to invade the host cell, and triggers an inflammatory response [8]. These systems appear to work synergistically in order to facilitate the colonization of the ileum. Meanwhile, the biofilm formation of *Salmonella* is closely related to its fimbriae. The structural genes *fimA*, *fimI*, *fimC*, *fimD*, *fimH*, and *fimF* are all expressed in one transcript from the P*_fimA_* promoter. FimH, as a lectin-like protein, is directly involved in binding to high-mannose oligosaccharides carried by the surface glycoproteins of eukaryotic cells [9]. There is a growing body of literature that recognizes that minor differences in the structure of FimH are most likely associated with differences in adhesion specificities and may determine the tropism of various *Salmonella serovars* to different species and tissues [10,11,12]. T1F is environmentally regulated, with fim gene expression favored in static liquid medium, whereas growth on solid medium inhibits expression [13]. Its main subunit is fimA, and multiple copies of fimA constitute the main body of the fimbriae. In serovar Typhimurium, the expression of the structural genes is regulated by three transcription factors, *fimY*, *fimZ*, and *fimW* [14]. Both *fimZ* and *fimY* are essential for the expression of the structural genes from the P*_fimA_* promoter, In particular, the deletion of either the *fimY* or *fimZ* gene prevents serovar Typhimurium from making T1F. *FimZ* has been shown to bind the P*_fimA_* promoter and promote transcription [15]. *FimY*, on the other hand, is thought to facilitate the activation of the P*_fimA_* promoter, as direct binding has not been observed. *FimW* is a negative regulator of *fim* and antagonizes with *fimZ* [16]. Fim can cause a strong intestinal inflammatory response. Studies have shown that the expression of inflammatory factors IL-1β and IL-6 in cells is higher than that of fimbriae-deficient strains after infection with wild-type *Salmonella* [17]. In the early stage of *Salmonella* infection, the inflammatory reaction helps the bacteria to obtain nutrients and compete with intestinal microbes. The pathogenicity of *Salmonella* is closely related to the condition of its fimbriae.

The natural antibacterial compounds applied to pathogenic microorganisms have received new attention due to the toxicity of synthetic chemicals and the concern for emerging strains of antibiotic-resistant bacteria. Plant-derived essential oils are a class of natural antibacterial agents that have traditionally been used as dietary ingredients, especially for preserving foods and enhancing food flavors. The antibacterial properties of several plant-derived essential oils have now been demonstrated [18]. Cinnamaldehyde, an aromatic aldehyde, is the main component of cinnamon bark extract and reported to have antibacterial activity against a variety of foodborne pathogens [19]. Cinnamaldehyde has a good inhibitory effect on various bacteria and fungi and inhibits the formation of biofilms [20]. In addition, studies have shown that cinnamaldehyde can downregulate the expression of bacterial virulence factors [21].

Until now, there is no study on the role of cinnamaldehyde on *Salmonella* fimbriae expression. In this study, the mechanism of cinnamaldehyde to inhibit T1F of *S*. typhimurium was evaluated using transmission electron microscopy, biofilm assays, hemagglutination inhibition tests, proteomics technology, real-time reverse transcriptase PCR, Western blotting and cell adhesion invasion assays.

## 2. Materials and Methods

### 2.1. Bacterial Strains, Cell Line, Animals and Reagents

*Salmonella enterica* serotype Typhimurium (ATCC14028) was purchased from the American Type Culture Collection (ATCC). The human colon carcinoma (Caco-2) cell was obtained from the College of Veterinary Medicine—Sichuan Agricultural University and cinnamaldehyde (purity ≥ 99.5%) was purchased from the Chengdu Herbpurify Co., Ltd. (Chengdu, China). The compound was dissolved in dimethyl sulfoxide (DMSO, ≥99.5%; Sigma, Shanghai, China) for experiments. One-day-old Roman chickens, negative for *Salmonella* by fecal culture and serological screening, were purchased from Chengdu Muxing Poultry Co., Ltd. (Chengdu, China) All chickens were adaptively reared in the Animal Room of Wenjiang Campus of Sichuan Agricultural University for 3 days.

### 2.2. Susceptibility Testing

Minimum inhibitory concentration (MIC) is an index to measure the antimicrobial activity of antimicrobial agents. It refers to the minimum concentration of drugs that can inhibit the growth of pathogenic bacteria in a culture medium after 18 h to 24 h in vitro. The minimum inhibitory concentration (MIC) of cinnamaldehyde against *Salmonella typhimurium* was determined by microbroth dilution according to CLSI 2015 guidelines [22]. The test was repeated three times at 37 °C.

### 2.3. Bacterial Growth Curve Assay

*Salmonella* was cultured statically in Tryptic Soy Broth (TSB) medium at 37 °C until *OD*_600nm_ = 0.3 then equally divided into five Erlenmeyer flasks. Cinnamaldehyde was added to each flask to obtain final concentrations of 0, 16, 32 and 64 μg/mL. Subsequently, the flasks were cultured at 37 °C. Other conditions were unchanged, and DMSO was used as a control group. The absorbance of the culture at different growth times was measured by using a HACH DR6000 UV VIS Spectrophotometer.

### 2.4. Hemagglutination Inhibition Test

*Salmonella* was inoculated in TSB medium and cinnamaldehyde was added to give a final concentration of 0, 16, 32 and 64 μg/mL. After 18 h at 37 °C, the culture was centrifuged (4000 rpm), washed twice with sterile PBS; the final concentration of the bacterial suspension was 1 × 10^10^ CFU/mL. Rabbit blood without fibrin was taken, washed with sterile PBS three times, to prepare 1% rabbit red blood cell suspension; 25 μL of bacterial suspension and rabbit red blood cells were dripped into a 96-well hemagglutination plate and then let stand for 30 min at room temperature to observe the agglutination results. The same procedure was used for the slide agglutination test, but for 2 min. The experiment was repeated three times.

### 2.5. Biofilm Assay

*Salmonella* was inoculated in TSB medium for 18 h at 37 °C and cinnamaldehyde was added to make a final concentration of 0, 16, 32 and 64 μg/mL. Other conditions were unchanged, and DMSO was used as a control group. The production of biofilm was determined by crystal violet staining [21]. Remove the medium and suspended bacteria, add 100 μL of 10% formaldehyde solution, incubate overnight at room temperature, and fix the biofilm. Formaldehyde was removed and washed three times with sterile PBS, and then 100 μL of 0.1% crystal violet (CV) was added to stain the biofilm. After incubation at 20 °C for 30 min, it was rinsed with ultrapure water and dried. Then, 200 μL 33% acetic acid was added to each well, and the absorbance *OD*_490nm_ was measured by a microplate reader. The experiment was repeated three times and averaged.

### 2.6. Transmission Electron Microscope (TEM)

*Salmonella* was inoculated in TSB medium and cinnamaldehyde was added to give a final concentration of 0, 16, 32 and 64 μg/mL. DMSO was added to the control group. After the bacteria were incubated at 37 °C for 18 h, the bacterial suspension was taken and centrifuged (1000 rpm, 5 min). After shaking again, 20 μL of bacterial liquid was absorbed on the wax plate, and the copper mesh supporting film was lightly covered over the bacterial liquid and let stand for 2 min; the excess bacterial liquid was dried with filter paper. Then 20 μL phosphotungstate dyeing solution was dropped onto the wax plate and covered with the copper mesh. After 50 s, the excess dyeing solution was blotted up with filter paper. The stained copper mesh was dried at room temperature and examined by transmission electron microscope (H-7650, Hitachi, Itd., Tokyo, Japan). Three to five bacteria were selected from each sample for observation. The experiment was repeated three times.

### 2.7. RNA Isolation and Reverse Transcription Polymerase Chain Reaction (RT-PCR)

The expression levels of *fimA*, *fimZ*, *fimY*, *fimH* and *fimW* were detected by real-time reverse transcription PCR. *Salmonella* was inoculated in TSB medium for 18 h at 37 °C, and cinnamaldehyde was added to make a final concentration of 0, 16, 32 and 64 μg/mL. DMSO was added to the control group. Total bacterial RNA was extracted according to the instructions of the total bacterial RNA extraction kit (Takara). The purity of RNA was measured for purity by a UV spectrophotometer (Agilent Technologies) at 260 nm and then transcribed into cDNA according to the instructions of a reverse transcription kit (Takara). PCR amplification and gene expression were assessed by the Real-Time System (CFX Connect™, Bio-Rad Laboratories, Inc, Hercules, CA, USA). All the above operations were performed on ice. The results were normalized to 16S RNA expression as an internal standard and calculated (Livak and Smith TGEN, 2001). The primer sequences used are shown in Table 1. The experiment was repeated three times.

### 2.8. Western Blotting

Western blotting was used to assess the content of T1F in TSB medium that was co-cultured with DMSO or cinnamaldehyde. The bacteria were cultured at 37 °C for 18 h. Then, the culture was centrifuged (6000× *g*, 4 °C, 10 min) and washed twice with sterile PBS. The bacterial suspension was broken with ultrasonic cell crushing apparatus (SM-650A, SHUNMA, Nanjing, China) and centrifuged (6000× *g*, 4 °C, 10 min) again to obtain the supernatant. The protein content in the supernatant was determined by a BCA kit (Jiancheng, Nanjing, China) to make sure each group contains the same protein concentration. An equal amount of three times the volume of the supernatant was added to one volume of a 4× sample buffer with 98 °C denaturation for 5 min. Supernatant samples (20 μL) were degraded with Laemmli SDS sample buffer and loaded onto sodium dodecyl sulfate (SDS)-polyacrylamide (12%) gel. After electrophoresis, proteins were transferred to polyvinylidene fluoride membranes and probed using a primary anti-T1F antibody and a secondary anti-rabbit antibody (HRP Goat Anti-Rabbit IgG).

The antibodies used in immunoblotting were as follows: anti-T1F antibody (1:2000; GenScript Biotech, Nanjing, China), and anti-rabbit secondary antibodies (1:2000; GenScript Biotech, China). T1F was detected using ECL western blotting detection reagents (Bio-Rad, ChemiDoc™ MP).

## 3. Proteomic Sample Preparation, Enzymolysis and Labeling Peptide Fragments

Bacteria (*OD*_600_ = 0.3) were cultured in TSB with DMSO or cinnamaldehyde (1/2 MIC) at 37 °C for 6 h. The bacterial cultures were centrifuged to collect the bacterial pellets, which were washed three times with phosphate-buffered saline to remove the residual culture medium. The pellets were then resuspended in a buffer containing 1% dithiothreitol (DTT) and a protease inhibitor. The cells were lysed with a sonic oscillator (Biosafer150-93, Chengdu, China) at 20 °C for total protein extraction. The lysate was then centrifuged at 12,000× *g* for 10 min at 4 °C. The supernatant was harvested for iTRAQ analysis.

A portion (400 μg) of each sample was subjected to enzymolysis with 100 mM DTT and then boiled for 5 min. The cooling samples were separated at several centrifugal velocities and divided into peptides of different lengths. The peptide fragments were labeled according to the instructions for the iTRAQ Reagent-6plex Multiplex Kit (AB Sciex, Framingham, MA, USA). The samples were dissolved in a labeling reagent containing 50 μL of isopropanol at room temperature for 1 h. All the labeled peptides were pre-classified with the AKTA Purifier 100 (GE Healthcare, Chicago, IL, USA), freeze-dried, and desalted. Then LC-MS/MS mass spectrometry was performed to check the iTRAQ labeling efficiency [23].

### 3.1. Proteomic Data Analyses

The protein information was obtained from the UniProt online database (http://www.uniprot.org/) accessed on 31 January 2018. The metabolic pathways or signal pathways in which the proteins participated were analyzed according to the Kyoto Encyclopedia of Genes and Genomes (KEGG) pathways (http://www.genome.jp/kegg/) accessed in 31 January 2018. Differentially expressed proteins were mapped to certain terms in the Gene Ontology database (http://www.geneontology.org/) accessed in 31 January 2018. The enrichment analysis was used to determine the most significant GO or KEGG pathways for the differentially expressed proteins.

### 3.2. Cytotoxicity and Cell Invasion Assay

Caco-2 cells were cultured in Dulbecco’s modified Eagle’s medium (DMEM; HyClone) supplemented with 10% fetal bovine serum (FBS; Biological Industries, Israel) and penicillin-streptomycin (except for infection assays) at 37 °C in a 5% CO_2_–humidified atmosphere. The cytotoxicity assay was measured with a cytotoxicity assay kit (CCK8, Transgen, Beijing). The results were measured at 450 nm absorbance with a microplate reader (Thermo, Waltham, MA, USA).

For the invasion assay, *Salmonella* (*OD*_600_ = 0.6) were washed in PBS, resuspended in cell culture medium and adjusted by dilution to provide an MOI (multiplicity of infection) of 10:1 bacteria to cells in culture wells of a 6-well plate. Confluent monolayers were infected for 1 h, followed by an additional hour of incubation with culture media containing 50 μg/mL of gentamicin (Sigma). After incubation, cells were washed three times with PBS and lysed with 0.1% Triton X-100 (Sigma) for 10 min. Bacterial suspensions were serially diluted with PBS, plated on LB-agar plates, and incubated overnight at 37 °C, and the colonies were then counted to calculate the CFU. The experiment was repeated three times and averaged.

### 3.3. Determination of Bacterial Load in Cecum

Chicks were randomly divided into a control group, a *Salmonella* challenge group, and a cinnamaldehyde group, with 30 chicks in each group. The challenge group and treatment group were intraperitoneally injected with 0.3 mL *Salmonella* PBS solution (3 × 10^8^ CFU/mL), and the control group was inoculated with the same amount of PBS solution. At 6 h after the challenge, chicks were given 100 mg/kg of cinnamaldehyde through oral administration, once a day.

The cecal flora was detected by the plate counting method. On 1, 3, and 5-days post infection (DPI), six broilers were slaughtered from each group, 0.5 g of cecal chyme was put into a sterilized bottle, and then 4.5 mL of normal saline was added into the bottle. After shaking for 2 min and standing for 10 min, a serial dilution was performed step by step in this way, with three replicates for each dilution degree. They were inoculated on SS agar plates and cultured for 24 h in a biochemical incubator at 37 °C for colony count.

### 3.4. Intestinal Factor Detection

On the 1, 3 and 5 DPI, six chicks were randomly selected from each group. Some intestinal jejunum tissues were taken out and put into EP bags, frozen in liquid nitrogen and stored at −80 °C for subsequent use. The sequences of tested genes in GenBank were searched. Primers were designed and analyzed using Primer Premier 5 software, and the best primer sequences were selected for subsequent experiments (Table 2). After the primer design was completed, it was purified and synthesized by Chengdu Qingke Biotechnology Co., Ltd. The detection of intestinal inflammation and immune factor gene expression was conducted by RT-PCR using β-actin as a reference gene.

### 3.5. Statistical Analysis

GraphPad software was applied for statistical analysis. Means and standard deviations were calculated and results were analyzed for statistical significance using Student’s *t*-tests for pairwise comparisons. *p* values  <  0.05 were considered statistically significant. The data of bacteria extracted from chicken cecal tissue were analyzed using two-way ANOVA.

## 4. Results

### 4.1. Cinnamaldehyde Inhibits the Expression of S. typhimurium fimbriae

**Figure 1:**
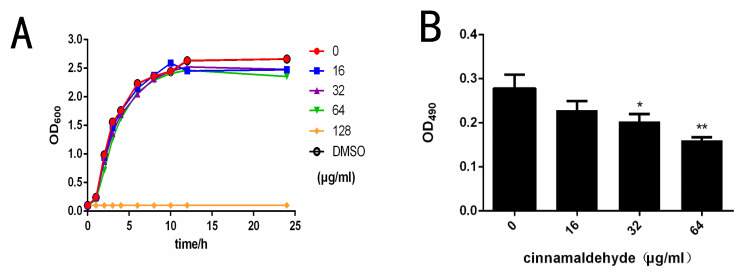

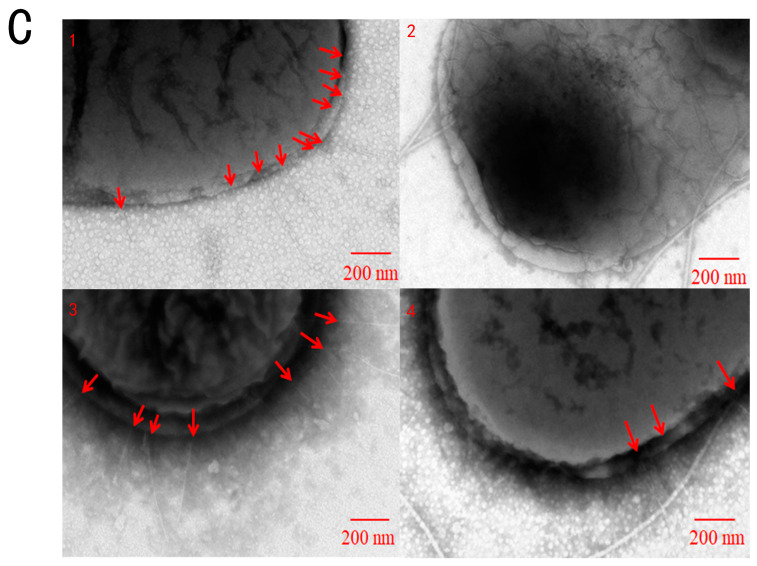
Cinnamaldehyde inhibits *Salmonella* type I fimbriae. (**A**) Growth curve of *S.* typhimurium co-cultured with different concentrations of cinnamaldehyde at 37 °C. (**B**) Effect of cinnamaldehyde at subinhibitory concentrations on growth of *S.* typhimurium biofilm. (**C**) Morphological effects of cinnamaldehyde at subinhibitory concentration on fimbriae: (**1**) 0 μg/mL group; (**2**) 16 μg/mL group; (**3**) 32 μg/mL group; (**4**) 64 μg/mL group. The red arrow indicates Type I fimbriae. Note: * means *p* < 0.05, ** means *p* < 0.01, compared with 0 μg/mL group.

The minimal inhibitory concentration of cinnamaldehyde against *S.* typhimurium was 128 μg/mL. The bacterial growth curve indicated that cinnamaldehyde had little effect on the growth of *S.* typhimurium under the minimum inhibitory concentration (Figure 1A). The results showed that the tested concentration of cinnamaldehyde in this study had no influence on the growth of *S.* typhimurium.

The erythrocyte agglutination ability of *Salmonella* co-cultured with different concentrations of cinnamaldehyde was measured. We noticed that the agglutination activity of *S.* typhimurium was negatively correlated with cinnamaldehyde concentration, as blood coagulation decreased significantly with increasing cinnamaldehyde concentration (Table 3). In contrast, as the drug concentration drops to 0, blood clotting becomes quite noticeable. The effects of cinnamaldehyde on hemagglutination could be related to a change in type I fimbriae expression. Fimbriae are involved in biofilm formation, so the effects of cinnamaldehyde on *Salmonella* biofilm formation on plastic were assessed. The results of biofilm formation showed that cinnamaldehyde significantly inhibited the formation of *S.* typhimurium biofilm at 32 and 64 μg/mL (Figure 1B).

The results of TEM showed that cinnamaldehyde had an inhibitory effect on fimbriae (Figure 1C). As can be seen from Figure 1, the negative group (Figure 1(C1)) had a large amount of T1F, and the distribution was relatively uniform. The fimbriae of the 16 μg/mL group (Figure 1(C2)) almost disappeared, and flagella were observed. This may be due to the phase variation of bacteria. *S.* typhimurium exists in two different phenotypes, adhesive and non-adhesive. Adherent cells have been shown to produce type I fimbriae. Conversion between the two phenotypes is called phase variation (Patterson, Borewicz et al. 2012). The number of pili decreased in the 32 μg/mL group (Figure 1(C3)) and 64 μg/mL group (Figure 1(C4)). These results indicated that cinnamaldehyde could reduce the expression of S. fimbriae expression without inhibiting the growth of bacteria.

### 4.2. Cinnamaldehyde Affects S. typhimurium Type I Fimbriae Related Factors

**Figure 2:**
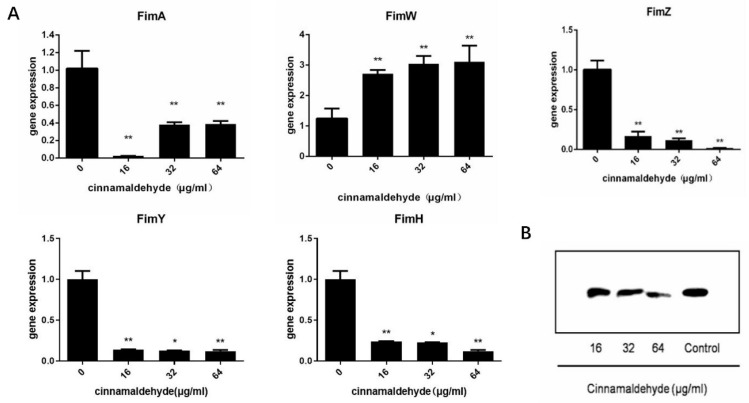
Cinnamaldehyde affects the transcription level of T1F (**A**) Transcription of expressed type I fimbriae opron genes of *S.* typhimurium co-cultured with different concentrations of cinnamaldehyde. (**B**) Western blot image of FimA content. Note: * means *p* < 0.05, ** means *p* < 0.01, compared with 0 μg/mL group.

Type I fimbriae is the only fimbriae expressed during static culture and the most important externally secreted fimbriae of *S.* typhimurium. The bacterial total RNA was extracted from the drug-treated group and the negative control group, and cDNA was reverse-transcribed. The expression of the target gene was detected by real-time fluorescence quantitative detection using cDNA as a template. The relative expression of each gene was calculated by the 2^−ΔΔCt^ method. The result is shown in (Figure 2A). As can be seen from the figure, the transcriptional levels of *fimW* were significantly increased after treatment with cinnamaldehyde, and the higher the cinnamaldehyde concentration, the greater the increase in transcriptional levels. The transcription level of *fimA*, *fimZ, fimY* and *fimH* was decreased after treatment, and the relative expression levels were <1. Under the action of cinnamaldehyde at low concentration, the expression level of *fimW* increased, and the relative expression level >2.

FimA is the main structural protein of type I fimbriae, accounting for more than 95% of the total protein of type I fimbriae. Western blotting analysis was used to detect the secretion levels of FimA in bacterial culture. We observed that the amounts of FimA were reduced by cinnamaldehyde treatment in a concentration-dependent manner (Figure 2B). Compared to the negative control group, we noted a decrease in FimA production at concentrations of 32 and 64 μg/mL cinnamaldehyde.

### 4.3. Cinnamaldehyde Effects the Proteomic Changes of S. typhimurium

Of the 2212 proteins detected with iTRAQ, 2057 showed no marked difference in their expression with or without cinnamaldehyde treatment. However, the expression of 73 proteins was upregulated and that of 82 proteins was downregulated. Proteomic analysis showed that the expression levels of six important virulence factors were significantly downregulated after cinnamaldehyde treatment (Table 4), including the fimbriae subunit (fimA and CR079_01855) and fimbriae regulator (fimW). SipA is the effector protein of *Salmonella* SPI-1, which can help *Salmonella* enter the host intestinal epithelial cells by endocytosis. InvB is the molecular chaperone of SipA, which assists in the folding and release of SipA in *Salmonella*. PrgI is a subunit protein of the SPI-1 pinhead protein, which can cause a host inflammatory response and help bacteria invade the host body.

**Figure 3:**
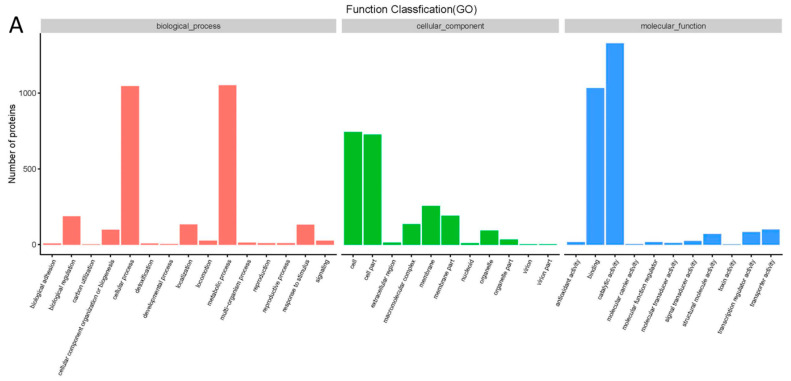

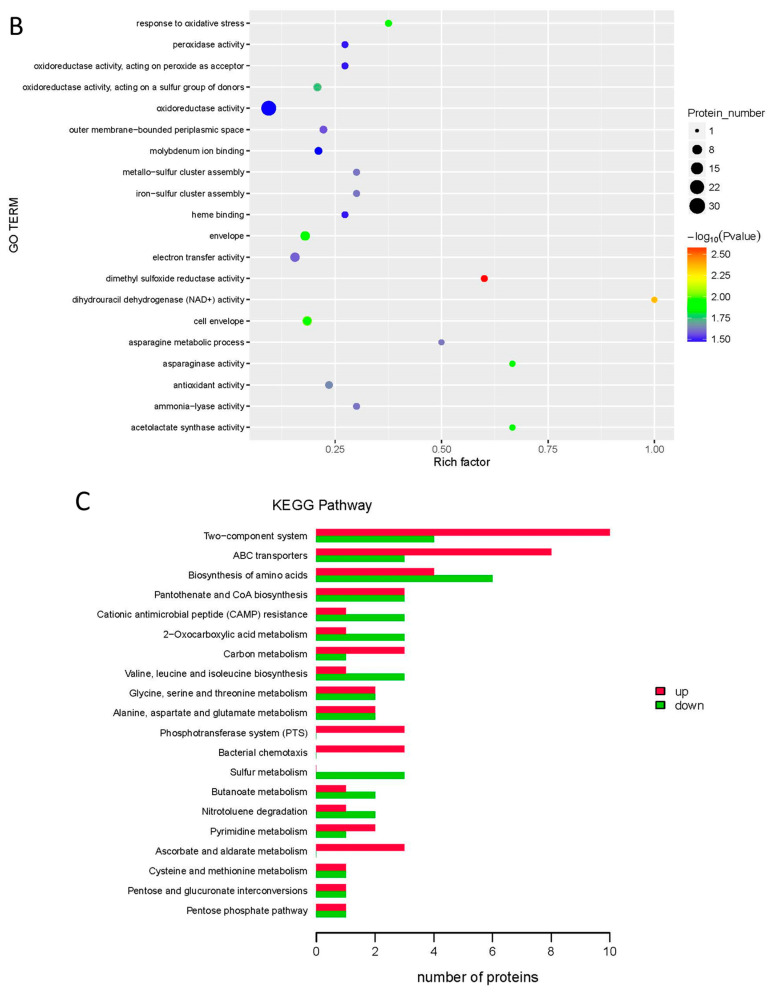

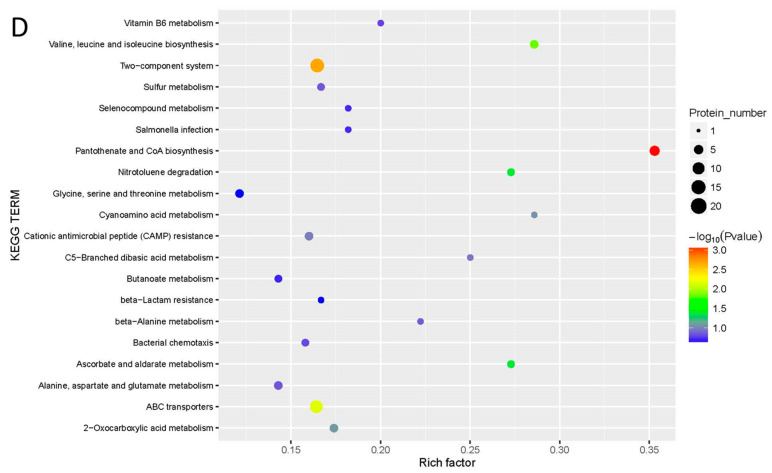
Effect of cinnamaldehyde on proteome of *Salmonella* (**A**) GO analysis of differently expressed proteins. (**B**) GO enrichment bubble diagram of differently expressed proteins. (**C**) KEGG pathway analysis of differently expressed proteins. (**D**) KEGG pathway enrichment bubble diagram of differently expressed proteins.

All the proteins detected with iTRAQ analysis were annotated to the three functional (GO) groups: biological process (BP), cellular component (CC), and molecular function (MF). The data are shown in Figure 3A. The proteins were annotated to 13 BP terms, 10 CC terms, and 9 MF terms. The differentially expressed proteins were also annotated to three functional (GO) groups. A GO enrichment bubble diagram is shown in Figure 3B. Most of the differentially expressed proteins were classified into several terms, such as cellular process and metabolic process in BP, cell and cell part in CC, and binding and catalytic activity in MF.

As shown in Figure 3C, 73 proteins were upregulated and 68 downregulated by cinnamaldehyde, and all the differentially expressed proteins were divided into 57 KEGG pathways. Figure 3D shows that the proteins were enriched in 20 KEGG pathway terms. The up- and downregulated proteins were mainly associated with the two-component system, ABC transporters, amino acids, pantothenate, and CoA biosynthesis.

### 4.4. Cinnamaldehyde Inhibited Salmonella Adhesion and Attenuated Intestinal Inflammation by Inhibiting Type I Fimbriae

**Figure 4:**
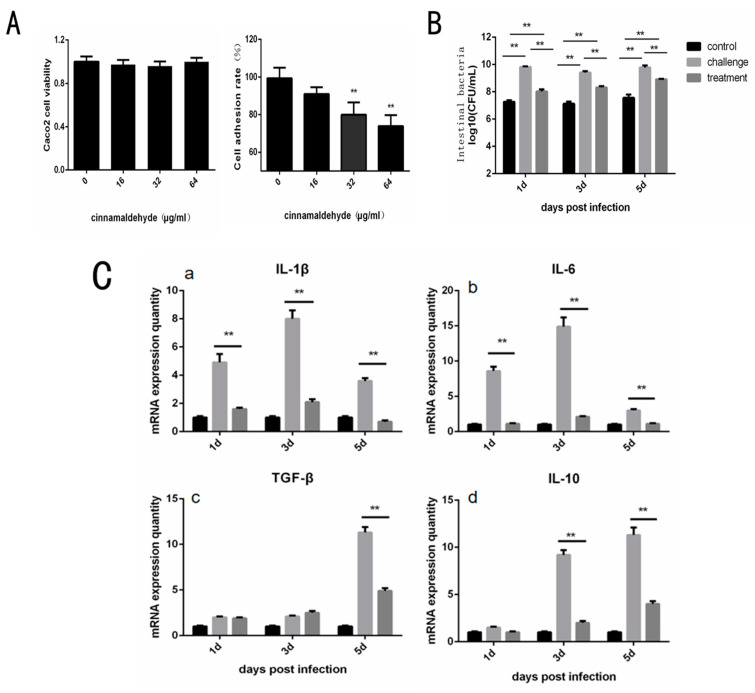

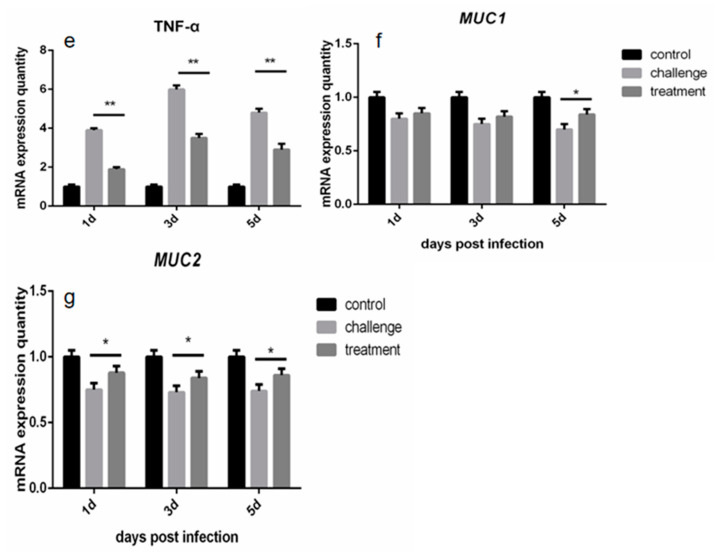
Effect of Cinnamaldehyde on Adhesion Cells of *Salmonella* and Protection of Chicken. (**A**) Cinnamaldehyde inhibits the adhesion of *S.* typhimurium to Caco-2 cells: Caco-2 cell viability and Cell adhesion rate of *S.* typhimuriumco-cultured with different concentrations of cinnamaldehyde. (**B**) Effects of cinnamaldehyde on cecal bacterial load of Salmonellosis chicks. (**C**) (**a**–**e**). Effect of cinnamaldehyde on the expression level of intestinal inflammatory factors (IL-1β, IL-6, IL-10, TGF-β and TNF-α) in chicks with salmonellosis. (**f**,**g**). Effect of cinnamaldehyde on intestinal mucosal immune factors MUC1 and MUC2 in chicks with salmonellosis. Note: * means 0.01 < *p* < 0.05, ** means *p* < 0.01.

By CCK-8 kit detection, it can be found that the concentration of cinnamaldehyde used in this experiment does little damage to Caco-2 cells (Figure 4A). In the adhesion test, the adhesion rate was expressed as the ratio between the number of adherent bacteria and the number of original bacteria, which was expressed as a percentage. Compared to 0 μg/mL, the cell adhesion rate in the treatment group with cinnamaldehyde decreased significantly (Figure 4A). These results suggest that cinnamaldehyde may inhibit the adhesion ability of *Salmonella* by inhibiting the Type I fimbriae.

The cecal bacterial load of chicks in different groups was measured by plate counting, and the results were shown in Figure 4B. At 1, 3 and 5 days, cecal bacteria in the challenge group increased by 35.12% ± 0.83%, 32.08% ± 1.02% and 29.37% ± 0.76%, respectively, compared to the control group. Compared to the control group, the cecal bacterial load in the treatment group increased by 10.50% ± 1.09%, 16.74% ± 0.89% and 17.95% ± 0.72%. Within 5 days, the intestinal bacterial load of chicks in the cinnamaldehyde treatment group was significantly decreased compared to in the challenge group (*p* < 0.01). These results suggest that cinnamaldehyde treatment does not confer intestinal resistance to *Salmonella* but reduces *Salmonella* colonization of cecal tissues in chicks.

Gene expression levels of intestinal inflammation and immune factors in chicks were obtained by RT-PCR reaction (Figure 4C). The results showed that at the initial stage of infection, the expression levels of intestinal inflammatory factors such as IL-1β, IL-6, IL-10, TGF-β and TNF-α in the treatment group were much lower than those in the challenge group (*p* < 0.01). These results indicate that cinnamaldehyde treatment can effectively reduce the expression level of intestinal inflammatory factors, and alleviate the acute inflammatory response caused by *Salmonella*. At the same time, the MUC1 marker is significantly higher after 5 days of treatment and MUC2 is significantly higher across all time points compared to the challenge group, suggesting that cinnamaldehyde can promote the intestinal mucosal immunity of chicks, and thus play a role in protecting the intestinal barrier.

## 5. Discussion

*Salmonella* is a gram-negative bacterium that is widely found in the gut of humans and animals. Currently, antibiotics are still the most commonly used treatment for *Salmonella*. Long-term use of these antibiotics may be an important reason for increased global bacterial resistance. In the past few years, there have been only a very small number of new drugs developed for gram-negative bacteria. Clinically, most strategies to treat resistant bacteria are to use multiple antibiotics or higher doses of antibiotics, which has led to the further development of multiple drug-resistant bacteria and more expensive treatment costs [24]. Therefore, the development of classical antimicrobial agents that directly target the pathogen must continue, but there is an urgent need for new therapeutic strategies to treat *Salmonella* infection.

Plant essential oils now have now received intense attention, such as the inhibition of biofilm formation, toxin production, bacterial quorum sensing, and adhesion factors [25]. In recent years, studies have been reported on the effects of paeonol, oleanolic acid, quercetin, magnolol and other natural plant active ingredients against pathogenic bacteria [26,27,28,29]. Cinnamaldehyde, an aldehyde compound, exists in the spice plant cinnamon. It is extensively used in the fields of medicine, food and cosmetics, and has properties of anti-inflammation, antioxidant, anti-bacteria and lowering blood pressure [30,31,32]. It was reported that cinnamaldehyde could exhibit antimicrobial properties in vitro against Enterobacteriaceae, such as *Escherichia coli*, *Salmonella enterica* and so on [33,34].

It is well known that fimbriae or pili play an important role in *Salmonella* biofilm formation and adhesion invasion. Studies have reported that cinnamaldehyde may inhibit the formation of enterohemorrhagic Escherichia coli O157:H7 biofilm and the ability to adhere to host cells mainly by inhibiting the production of pili [35]. Type I fimbriae (T1F), as an important virulence factor of *Salmonella* pathogenicity, could adhere to host epithelial cells and regulate biofilm formation [36].

In order to better understand the effect of *Salmonella* on T1F, we conducted a series of studies. In susceptibility testing, cinnamaldehyde showed a certain antibacterial effect on *S.* typhimurium in vitro (MIC = 128 μg/mL). However, in vivo, cinnamaldehyde can hardly reach an effective inhibitory concentration. Surprisingly, clinically, Salmonellosis in laying hens can be effectively treated by ingesting cinnamaldehyde [21], suggesting that cinnamaldehyde does not treat diseases by killing *Salmonella* in vivo, but by other mechanisms.

The typical manifestation of the T1F of *S.* typhimurium is the ability to agglutinate red blood cells [37]. We co-cultured *Salmonella* with different concentrations of cinnamaldehyde and took a hemagglutination test. The results showed that cinnamaldehyde could weaken the agglutination ability of *Salmonella*. The hemagglutination test confirmed the inhibitory effect of cinnamaldehyde on T1F. Similarly, The production of *salmonella* biofilms is closely related to the activity of T1F. Biofilm experiments proved that cinnamaldehyde significantly inhibited the formation of *S.* typhimurium biofilms at 64 and 32 μg/mL. This provides a hypothesis that the decrease in the production of *Salmonella* biofilms may be related to the inhibition of T1F by cinnamaldehyde.

T1F is the only expression of pili in static culture and can bind to mannose residues of the host intestinal epithelial membrane to mediate *Salmonella* adhesion, thereby promoting the formation of microcolonies and assisting bacterial colonization in the host intestinal tract. Adhesion to host tissues involves a specific interaction between pathogen and host proteins expressed at the surface of the cells. FimA is the main structural subunit of *Salmonella* type I pili encoded by the FIM gene cluster. FimW is a negative regulator of pili, and CR79_01855 is involved in pili assembly. After cell adhesion to epithelial cells, SPI-1 translocates effector proteins into cells via the needle structure. Effector proteins cause membrane folds, allowing bacteria to enter cells in pinocytosis mode. PrgI is the main subunit of the needle structure, which is assembled to form a hollow cylindrical structure, and the effector protein is secreted to the bacterial membrane through the central pore. SipA binds to actin and InvB assists in SipA translocation.

Anna et al. demonstrated that bacterial type I fimbriae adhere to host epithelial cells and regulate biofilm formation, dependent upon the *fimH* gene [36]. The type I fimbriae of *S. enterica* bind to the host membrane plasminogen and convert plasminogen to plasmin. Here, we focus on T1F and its adhesin FimH, one of the most abundant Enterobacteriaceae adhesin structures. After the co-culture of cinnamaldehyde, the expression level of *fimW* was upregulated, while the expression levels of *fimY* and *fimZ* were downregulated, leading to the downregulation of the *fimA* gene in the fimbriae. As the major fimbria subunit, the expression of *fimA* can reflect the production of fimbriae [38]. The results showed that cinnamaldehyde inhibited T1F by up-regulating the negative regulator *fimW*. Subsequently, we found that the expression of *fimH*, a key adhesive of *Salmonella*, was also decreased after co-culture of cinnamaldehyde. In addition, Western blotting analysis also confirmed the inhibitory effect of cinnamaldehyde on the protein expression level of FimA, the main structure of type I fimbriae.

In cell biology, the regulation of proteins includes transcription, post-transcription, translation and post-translation. In our results, the transcription level of fimW in *Salmonella* was inconsistent with the protein expression level. The possible reasons are the following: (1) cinnamaldehyde affected directly the stability of FimW; (2) cinnamaldehyde inhibited the related amino acids of FimW in *Salmonella*; (3) cinnamaldehyde increased the permeability of the *Salmonella* cell membrane, leading to the stability of FimW.

When *Salmonella* enters the body, it can cause acute gastrointestinal inflammation, such as diarrhea and intestinal congestion, which can lead to sepsis and even death in severe cases. Studies have shown that cinnamaldehyde attenuated the inflammatory response, oxidative stress, and apoptosis in the liver of Salmonella typhimurium-infected mice [39]. T1F was considered important, as they frequently have been shown to mediate adhesion to host tissues and, in a few well-studied cases, to facilitate adhesion and colonization in the early stages of an infection. With the above verification study, we simulated the intestinal invasion of *Salmonella* through the Caco2 cell model. It was found that cinnamaldehyde at a concentration of 64 μg/mL and 32 μg/mL can reduce the rate of adherent cells of *Salmonella*. Secondly, through intestinal bacterial load detection, it was found that cinnamaldehyde effectively prevented *Salmonella* colonization in the cecum of chicks, which was also consistent with the results of cell tests of bacterial adhesion and invasion in vitro. The inflammatory response caused by *Salmonella* is accompanied by the release of various inflammatory cytokines. After treatment with 100 mg/kg cinnamaldehyde, the expression levels of intestinal inflammatory factors such as IL-1β, IL-6, IL-10, TGF-β and TNF-α in the treated group were significantly lower than those in the challenge group (*p* < 0.01). It has been shown that cinnamaldehyde can inhibit the excessive inflammatory response and thus protect the body against *Salmonella* colonization. We believe that cinnamaldehyde can antagonize the colonization of *Salmonella* in the intestine, which is caused by its inhibition of T1F. After that, we tested the cytotoxicity of cinnamaldehyde and found that its cytotoxicity was weak, which suggested that cinnamaldehyde provided a certain prospect as a supplementary therapeutic agent for antibiotics.

In this study, we directly or indirectly demonstrated the inhibitory effect of cinnamaldehyde on the T1F of *S*. typhimurium by various means. This research is only a preliminary discussion of why cinnamaldehyde can treat *Salmonella* infection. Further profound research on the effects of cinnamaldehyde on *Salmonella* pili will be carried out in the future. In summary, cinnamaldehyde is expected to become a new type of anti-bacterial auxiliary drug, which plays a role in the frontier of anti-*Salmonella* infection.

## Figures and Tables

**Table 1:** Primer sequences with their corresponding PCR product length.

Gene	Primer Sequence (5′-3′)	Tm (°C)	Product Length
16s-F	ATGGCTCAGATTGAACGC	53	117 bp
16s-R	GGCAGTTTCCCAGACATTAC		
fimA-F	TGCCTTTCTCCATCGTCC	53	134 bp
fimA-R	TGCGGTAGTGCTATTGTCC		
fimW-F	ATCAGCTACGGGCGATTA	50	169 bp
fimW-R	CCAGAAGGGACGCTATGT		
fimZ-F	GGCACCGACGGCTTTACCT	51	116 bp
fimZ-R	CCCGCTCTTATTGCTCTTCC		
fimY-F	CACGCAGGGAAAGACACC	53	293 bp
fimY-R	CGCCTCCATATCTACAATCAGT		
fimH-F	ATCCCTCGCCAGACAATG	53	155 bp
fimH-R	TCGCCGAAATCAAACTCC		

**Table 2:** Primer sequence of intestinal factor gene in chicks.

Gene	Primer (5′-3′)	Tm (°C)	Product
β-actin-F	GGTATGGGCCAGAAAGAC	56	165 bp
β-actin-R	CTCCTCACGGGCTACTCT		
IL-1β-F	GGTCAACATCGCCACCTACA	59	86 bp
IL-1β-R	CATACGAGATGGAAACCAGCAA		
IL-6-F	AAATCCCTCCTCGCCAATCT	59	106 bp
IL-6-R	CCCTCACGGTCTTCTCCATAAA		
IL-10-F	CGGGAGCTGAGGGTGAA	58	272 bp
IL-10-R	GTGAAGAAGCGGTGACAGC		
TGF-β-F	CGGGACGGATGAGAAGAA	58	141 bp
TGF-β-R	TCGGCGCTCCAGATGTAC		
TNF-α-F	TGTCGGTCAGCCGCTTCTC	62	219 bp
TNF-α-R	TGGTCGCCTCCAACTCGTC		
MUC1-F	TCGCCTTGGAGGAATCTA	55	395 bp
MUC1-R	AGCAGTGGCAATGGTATCT		
MUC2-F	TGAGTCAGGCATAAATCG	53	419 bp
MUC2-R	GGTCTAAGTCGGGAAGTG		

**Table 3:** Hemagglutination inhibition test.

Diluted Multiples	1:02	1:04	1:08	1:16	1:32	1:64	1:128	1:256
0 μg/mL	++	++	+	+	+	+	-	-
16 μg/mL	+	+	+	+	+	-	-	-
32 μg/mL	+	+	+	-	-	-	-	-
64 μg/mL	+	+	-	-	-	-	-	-
PBS	-	-	-	-	-	-	-	-

Note: ++ means clotting is clearly visible, + means slightly clotting, - means no clotting.

**Table 4:** Differently expressed proteins related to toxins.

Accession Name	Gene Name	Function	Fold Change	*p* Value
A0A0D6H7T1	fimA	Fim subunit	0.80	0.02
A0A0M2IX42	CR079_01855	Fim subunit	0.72	0.01
A0A0D6H693	fimW	Fim regulator	0.73	0.02
A0A0C5PU36	invB	SPI-1molecular chaperone	0.66	0.03
A0A0C5PPT0	sipA	SPI-1effector	0.61	0.0002
A0A0C5Q2B9	prgI	SPI-1needle protein	0.55	0.001

## Data Availability

The raw date supporting the conclusions of this article will be made available by the authors, without under reservation.
